# Selection and validation of appropriate reference genes for quantitative real-time PCR analysis in *Salvia hispanica*

**DOI:** 10.1371/journal.pone.0186978

**Published:** 2017-11-01

**Authors:** Rahul Gopalam, Sunny D. Rupwate, Ajay W. Tumaney

**Affiliations:** 1 Department of Lipid Science, CSIR-Central Food Technological Research Institute (CSIR-CFTRI), Mysore, Karnataka, India; 2 Academy of Scientific and Innovative Research (AcSIR), CSIR-Central Food Technological Research Institute (CSIR-CFTRI), Mysore, Karnataka, India; Michigan State University, UNITED STATES

## Abstract

Quantitative real-time polymerase chain reaction (qRT-PCR) has become the most popular choice for gene expression studies. For accurate expression analysis, it is pertinent to select a stable reference gene to normalize the data. It is now known that the expression of internal reference genes varies considerably during developmental stages and under different experimental conditions. For *Salvia hispanica*, an economically important oilseed crop, there are no reports of stable reference genes till date. In this study, we chose 13 candidate reference genes viz. Actin11 (ACT), Elongation factor 1-alpha (EF1-α), Eukaryotic translation initiation factor 3E (ETIF3E), alpha tubulin (α-TUB), beta tubulin (β-TUB), Glyceraldehyde 3-phosphate dehydrogenase (GAPDH), Cyclophilin (CYP), Clathrin adaptor complex (CAC), Serine/threonine-protein phosphatase 2A (PP2A), FtsH protease (FtsH), 18S ribosomal RNA (18S rRNA), S-adenosyl methionine decarboxylase (SAMDC) and Rubisco activase (RCA) and the expression levels of these genes were assessed in a diverse set of tissue samples representing vegetative stages, reproductive stages and various abiotic stress treatments. Two of the widely used softwares, geNorm and Normfinder were used to evaluate the expression stabilities of these 13 candidate reference genes under different conditions. Results showed that GAPDH and CYP expression remain stable throughout in the different abiotic stress treatments, CAC and PP2A expression were relatively stable under reproductive stages and α-TUB, PP2A and ETIF3E were found to be stably expressed in vegetative stages. Further, the expression levels of Diacylglycerol acyltransferase (DGAT1), a key enzyme in triacylglycerol synthesis was analyzed to confirm the validity of reference genes identified in the study. This is the first systematic study of selection of reference genes in *S*. *hispanica*, and will benefit future expression studies in this crop.

## Introduction

Chia (*Salvia hispanica* L.) is a flowering plant that belongs to the mint family (Lamiaceae) and is native to parts of South America and Mexico. It is an important oilseed crop, having about 60% α-linolenic acid (ALA) in its seed triacylglycerol and is the highest reported terrestrial plant source of this omega-3 fatty acid [1]. Besides ALA, the seeds of this plant are also a wealthy source of dietary fiber, protein, essential minerals and vitamins [2]. The transcriptome analysis has been performed recently on the developing seeds of *S*. *hispanica* and different enzymes have been identified that are proposed to play a pivotal role in the flux of ALA into triacylglycerol (TAG) [3]. To further our understanding of biosynthetic pathway leading to the accumulation of ALA in TAG, gene expression analysis is an important tool. Currently, there are no reports on internal reference genes in *S*. *hispanica*. Therefore, the exploration of reference genes for precise transcript normalization in seed development as well as under different experimental conditions is important for the future research to be carried out on *S*. *hispanica*.

Gene expression analysis has become an important tool in multiple fields of biological research. Real time quantitative reverse transcription PCR (also referred to as qRT-PCR) is now the most popular approach to measure the levels of gene expression; its main advantages being high sensitivity, efficiency, specificity and a broad quantification range [4,5]. For accurate analysis of gene expression, normalization of gene expression data with suitable internal reference gene(s) is needed [6]. Normalization is extremely important to correct the variations that might arise due to the quality and amount of template used, both of which can have an effect on the efficiency of reactions [7]. Some researchers have shown that two or more reference genes may be needed for data normalization [8,9]. This method requires the use of internal reference genes expressed constitutively to account for the variability seen at different steps of experimental procedure.

The most commonly used reference genes (also referred to as housekeeping genes) are those that are involved in basic and ubiquitous cellular processes such as the glycolytic pathway, ribosomal subunit synthesis, protein folding and components of cytoskeleton. Most of the traditionally used reference genes validated in plants include, glyceraldehydes-3-phosphate dehydrogenase *(GAPDH)*, ubiquitin *(UBQ)*, β-actin (*ACT)*, α-tubulin *(TUB)*, elongation factors *(EF)*,*and 18S* ribosomal RNA [10–14]. These genes are assumed to have constant expression levels between different samples and in response to different environmental stimuli. However, recent studies have shown that the expression levels vary under different experimental conditions in various tissues and organs [15–18]. Therefore, it is important to choose multiple stably expressed reference genes other than the traditional ones for the precise normalization of gene expression studies.

In the recent years, several new reference genes with stable expression have been identified as ideal genes such as, *F-box* protein in soybean [19], protein phosphatase 2A (*PP2A)* in buffalo grass [20] and Sorghum [21], clathrin adaptor complex subunit family protein (*CAC)* and TIP41-family protein in *Brassica juncea* [22]. Hence, it is important to choose the suitable reference genes across various experimental conditions in different species.

Many studies of stable reference genes have been reported in various plant species such as *Arabidopsis thaliana* [6, 23], *B*. *juncea* [22], *Oryza sativa* [16, 24], tea plant [25] tomato [26], *Glycine max* [27, 28], potato [11], Sorghum [21], flax [12], *Arachis hypogea* [29], *Triticum aestivum* [30], *Cajanus cajan* [31], *Zea mays* [32, 33] and so on. To the best of our knowledge, no studies have been carried out regarding the selection of stable reference genes in *S*. *hispanica*.

In the current study, we have analyzed the expression of 13 candidate reference genes in diverse tissue samples of *S*. *hispanica*, broadly categorized into three distinct tissue sets (abiotic stress treatments, vegetative stages and reproductive stages). Two statistical algorithms, geNorm [8], Normfinder [7], were performed to evaluate the expression stability of reference genes and to determine the optimal number of genes required for normalization in different tissue sets. Furthermore, the expression level of Diacylglycerol acyltransferase 1 (DGAT1) was checked using the stable reference genes identified in the present study to validate its effectiveness.

## Materials and methods

### Plant materials and stress treatments

Chia (*S*. *hispanica*) variety CHIAmpion B-1 was used for all the experiments. The samples have been categorized in three different experimental sets. Pre-germinated seeds on a filter paper saturated with water were grown in plastic pots in green house. Seedlings were assessed at the second true leaf stage and the healthy ones were used for i) abiotic stress group and ii) vegetative groups.

In the vegetative group, a total of 5 tissues namely, cotyledons, root, shoot, young leaf (before flowering) and mature leaf (after flowering) were collected from the plants. For stress treatments, seedlings at the second true leaf stage were transferred to separate beakers each containing 200 mM NaCl, 15% PEG 6000, 200 μM Salicylic acid (SA) and 100 μM abscisic acid (ABA) and were incubated for 5 h. For cold and heat treatments, seedlings in pots were placed in chambers at 4 ± 1°C and 42 ± 1°C respectively, for 5 h. Control plants were kept in room temperature at 25 ± 1°C. Leaves were collected from three independent biological replicates for each treatment.

In the reproductive group, a total of 6 tissues namely, bud, seed 3 days after flowering (DAF), seed 7 DAF, seed 14 DAF, seed 21 DAF and seed 28 DAF were collected from the plants grown in the green house. All tissues were immediately frozen in liquid nitrogen and stored at -80°C till further use.

### Total RNA isolation and cDNA synthesis

Total RNA was extracted using Spectrum Plant Total RNA Kit (Sigma, USA) following the manufacturer’s instructions. The integrity of RNA samples was verified by performing 1.5% (w/v) agarose gel electrophoresis and the quality was determined using Nanodrop 2000 Spectrophotometer (NanoDrop Technologies, Thermo Scientific, USA). The quantity was measured using Qubit 3.0 Fluorometer (Thermo Scientific, USA). Only the RNA samples with absorption ratios of A_260/280_ = 2.0–2.1 and A_260/230_ higher than 2.0 were used for cDNA synthesis. The integrity of all RNA samples was further measured using Agilent 2100 Bioanalyzer (Agilent technologies, Palo Alto, CA) and RIN (RNA integrity number) of > 6.5 were used for analysis (S1 File).

An aliquot of 1 μg of total RNA was used for cDNA synthesis with a final volume of 20 μL using Thermo Scientific Verso cDNA synthesis kit with RT Enhancer, to remove any contaminating DNA, by following the manufacturer’s instructions. The cDNA was diluted 1:10 with nuclease-free water for use in qRT-PCR.

### Selection of candidate reference genes and primer design

Based on previous literature reports of stable expression in different plant species, 13 candidate reference genes were shortlisted. The amino acid sequences of their corresponding homologs taken from TAIR (https://www.arabidopsis.org/) database served as a query for a TBLASTN search against assembled transcripts of *S*. *hispanica* [3]. Local blast (TBLASTN) was done using the software given in the following link (https://www.blaststation.com/intl/en/blaststation2.php). The best hit for each gene was chosen based on the bit score and E-value. The 13 candidate reference genes include *ACT11*, *EF1-α*, *ETIF3E*, *α-TUB*, *β-TUB*, *GAPDH*, *CAC*, *RCA*, *FtsH*, *PP2A*, *CYP*, *18S rRNA and SAMDC*. The candidate reference genes, their homologs in Arabidopsis and GenBank accessions are provided in Table 1. Primer pairs were designed using Primer3Plus (http://primer3plus.com/cgi-bin/dev/primer3plus.cgi) [34] based on the following criteria: melting temperatures of 58–62°C, primer lengths of 18–20 bp, GC content of 40–60%, and amplicon lengths of 100–150 bp. All the primer sequences in the current study are listed in Table 2. The specificity of each primer pair for its target was further confirmed by Primer-BLAST.

**Table 1:** Candidate reference genes and their GenBank accessions in *S*. *hispanica*.

Gene name (abbreviation)	GenBank accession number	Arabidopsis homolog locus	Arabidopsis TBLASTN E-value	Identity (%) to *A*. *thaliana*
Actin11 (*ACT)*	MF621045	AT3G12110	0.0	96%
Elongation factor-1alpha (EF1-α)	MF621042	AT5G60390	0.0	95%
Eukaryotic translation Initiation factor 3E (ETIF3E)	MF621041	AT3G57290	0.0	82%
Alpha tubulin α-(TUB)	MF621046	AT5G19780	0.0	87%
Beta tubulin β-(TUB)	MF579140	AT4G20890	0.0	90%
Glyceraldehyde-3-phosphate dehydrogenase (GAPDH)	MF574098	AT1G13440	1e-160	86%
Clathrin adaptor complex (CAC)	MF621044	AT5G46630	0.0	90%
Serine/threonine-protein phosphatase (PP2A)	MF579139	AT1G10430	1e-177	94%
Rubisco activase (RCA)	MF621043	AT2G39730	1e-165	85%
FtsH protease (FtsH)	MF621047	AT2G26140	0.0	74%
Cyclophilin (CYP)	MF579138	AT5G35100	1e-100	77%
Sadenosylmethionine decarboxylase (SAMDC)	MF579137	AT3G25570	1e-123	65%
18S ribosomal RNA (18SrRNA)	MF623893	AT3G41768	0.0	96%

**Table 2:** Comprehensive details of genes and primer sets used for qRT-PCR.

S. No	Gene Name	Primers (F/R) 5’-3’	Amplicon length (bp)	Amplicon Tm (°C)	PCR efficiency (E%)	(R^2^)
1	Actin11	ACTGGAATGGTCAAGGCTGG TCTTTCTGGCCCATCCCAAC	113	84.5	97.3	0.999
2	Elongation factor-1alpha	CTGTCCAGGAGCCAAAGAGG TCAACTCTTCCGACTGGCAC	103	82.5	104	0.999
3	Eukaryotic translation Initiation factor 3E	TGTGGAAACTACTCCGGTGC CTGCTGCCAATTTTCCCCAC	103	81	105.6	0.996
4	Alpha tubulin	CTCGCGCATTGACCACAAAT GAGCTTCGCTGAACTCACCT	101	82.5	104.3	0.999
5	Beta tubulin	GTACACCGGGGAAGGAATGG TTCGTCAGCAGTCGCATCTT	106	82.5	97.5	0.997
6	Glyceraldehyde-3-phosphate dehydrogenase	TCAAGGAGGAGTCTGAGGGA CAATTCCAGCCTTGGCATCG	120	81.5	109.7	0.995
7	Clathrin adaptor complex	CGGCTTCCGCGATTTACTTC GCACTCGAAAAGCATCCACC	102	83	99.9	0.999
8	Serine/threonine-protein phosphatase	GGCGATATCCACGGACAGTT CCGAATAGTACCCACGGTCG	115	82.5	94	0.999
9	Rubisco activase	CCGAAGATGACGCTGGAGAA AAGAGCTGCGTCCTTCAAGT	111	84	105.1	0.997
10	FtsH protease	GCGCCAAAGCAGTGAGTATG CTCGTGGTAGGCTGTCATCC	119	82	99.6	0.999
11	Cyclophilin	ACCAACCATTTCTTCCGGGT CTCAGCTTCCAACCTCTGCA	105	82.5	89.3	0.999
12	S-adenosyl methionine decarboxylase	GCTTCATATTCCCGGGTGCT TCCCATCACATAGGCTTCGC	116	83	89.3	0.998
13	18S ribosomal RNA	CGGGTGACGGAGAATTAGGG TACCTCCCCGTGTCAGGATT	111	84.5	109.5	0.993
14	^#^ Diacylglycerol acyltransferase1 (DGAT1)	GGAAGGGTTGGGTGGTAAGG CCCTCTCCACGGCATACAAA	144	78.5	99.2	0.999

The PCR efficiency (E%) and regression coefficient (R^2^) were calculated by the CFX96 system.

^#^ Used for normalization during reproductive stages.

### qRT-PCR analysis

qRT-PCR amplification was carried out on the Bio-Rad CFX96 Real-Time PCR system C1000 Thermal Cycler (Bio-Rad, Hercules, CA) using iQ SYBR Green Supermix (Bio-Rad, Hercules, CA). Each 10 μL reaction mixture included 3 μL of diluted cDNA, 5 μL of iQ SYBR Green Supermix, 0.5 μL each primer (0.5 μM) and 1 μL of Milli-Q H_2_O. Reaction conditions include an initial denaturation step at 95°C for 5 mins followed by 39 cycles of 95°C for 15 s, respective annealing temperatures for 30 s and 72°C for 30 s, after which a melt curve was generated at 55–95°C to confirm the specificity of each primer pair. Each qRT-PCR reaction was performed with three biological and technical replicates and a no-template (NTC) control was used.

To determine the specificity of primer pairs used in the current study, agarose gel electrophoresis and a melt curve analysis were performed. Firstly, a gradient was performed using mixed cDNA to determine the optimal annealing temperature of each primer pair (S1 Table). PCR amplifications were then carried out under the conditions; 94°C for 30 s, 39 cycles of 94°C for 30 s, respective annealing temperatures for 30s, 68°C for 30 s followed by a final extension step at 68°C for 5 min. Each sample with a total reaction volume of 100 μL contained 68.5 μL of Milli-Q H_2_O, 10 μL of Taq Buffer, 10 μL of dNTP mix, 5 μL of each primer (diluted to 10 μM), 0.5 μL of Taq Polymerase (New England Biolabs) and 1 μL of template. Amplified PCR products were run on a 2% agarose gel for confirmation. Products of correct size were gel extracted using Macharey-Nagel Gel Extraction Kit.

The gel purified DNA product concentration was measured by checking the absorbance at 260nm. The copy number was calculated by using the formula given in the following link (http://cels.uri.edu/gsc/cndna.html).

The purified DNA product was diluted with double distilled water to obtain a standard series differing by 10-fold. A series of 10 fold of five dilutions of cDNA (100–10,00,000) was used to determine the PCR efficiency of each primer pair by qRT-PCR. A standard curve was generated using linear regression and the slope based on the Cq values for all dilutions in a series. The qBaseplus software calculated the PCR efficiency using following equation:

$$Efficiency\;\%=10^{\left(\frac{-1}{slope}\right)}X\;100\%$$

### Data analysis

The quantification cycle (Cq) values of reference genes under different experimental conditions were recorded by CFX96 system. Two statistical programs, geNorm and Normfinder analysis were performed to rank the stability of suitable reference genes in different studied experimental conditions. The raw Cq values for all genes were corrected according to their PCR efficiencies and then changed to relative quantities. The relative expression values were then used in analysis to determine expression stability. geNorm calculates the gene expression stability value (*M*) based on pairwise variation (V) of a particular gene in comparison with all the other reference genes. The default set value is 1.5; gene with the lowest *M*-value is the most stably expressed one. Pair-wise variation analysis (Vn/n+1) was performed to decide the number of optimal reference genes needed for normalization in each sample set using qBaseplus software (v:3.0; Biogazelle, Belgium) [35]. A (Vn/n+1) value <0.15 indicates that no additional reference genes are needed for normalization. Normfinder measures the gene expression stability by estimating the intra- and inter-group variations. As with geNorm, the lower the stability value of the gene, the more stable it is.

### Reference gene validation

To validate the reliability of qRT-PCR data, the expression of a key target gene in triacylglycerol synthesis, DGAT1 was analyzed using the two most stable and a least stable genes identified in the study. The sequence of DGAT1 was extracted from *S*. *hispanica* transcriptome data and the amplification efficiency was calculated with qBaseplus software. The relative expression levels were calculated using qBaseplus software and represented as fold change [35].

## Results

### Verification of primer specificity and amplification efficiency analysis

**Fig 1:**
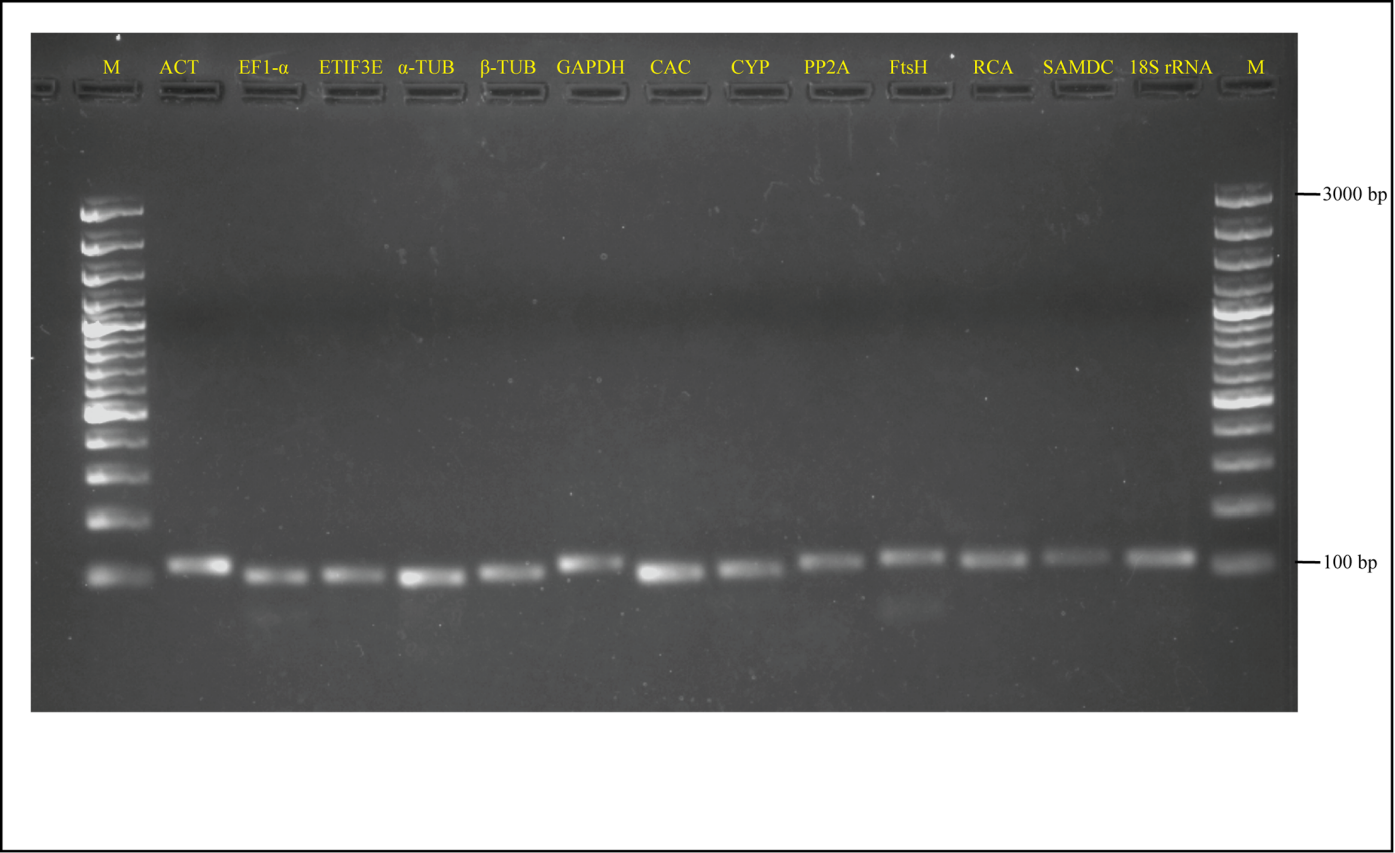
Agarose gel (2%) electrophoresis showing PCR amplified products of the expected size for 13 candidate reference genes. M: 100 bp marker (Thermo Scientific).

A total of 13 candidate reference genes representing different classes have been selected for this study. The gene names, amplicon length and Tm, amplification efficiency (E) and correlation coefficients (R^2^) are listed in the Table 2. All the primer pairs of 13 reference genes yielded a single PCR product of expected size upon amplification of pooled cDNA with no formation of primer dimers or non-specific amplification (Fig 1). Moreover, the melt curve analysis confirmed the specificity of amplicons as observed by the presence of a single peak and it also generated the melting temperatures (S2 File). In addition, the complete ORFs of all the genes were sequence verified and the amplicon sequences of the respective genes used in the current study are given in S2 Table. Amplification efficiencies of qPCR reactions ranged from 89.3% for CYP and SAMDC to 109.1% for 18S rRNA and correlation coefficients (R^2^) of the standard curve ranged from 0.993 for 18S rRNA to 0.999 for CAC (S3 File).

### Cq value analysis

**Fig 2:**
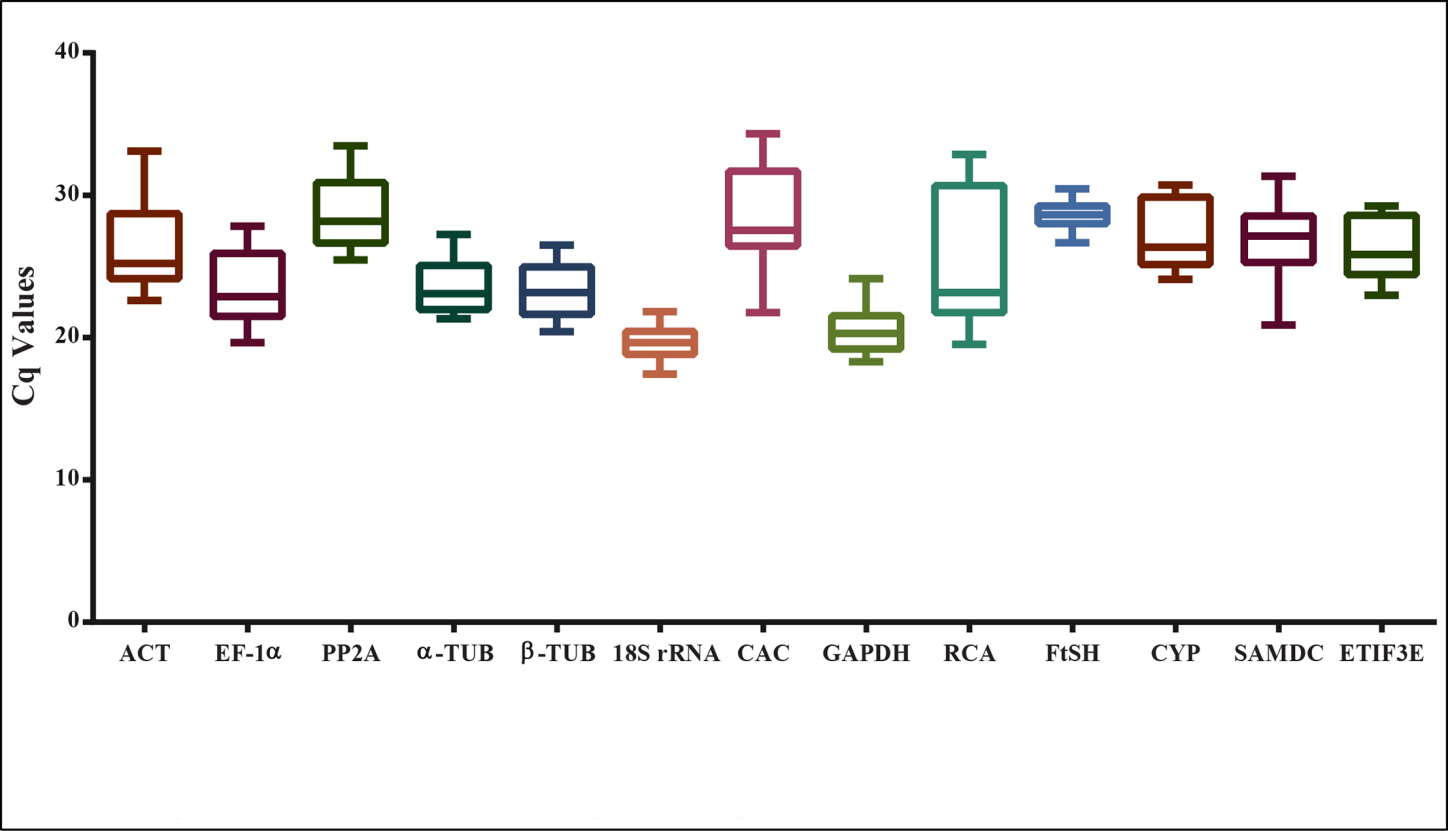
Distribution of quantification cycle (Cq) values across all experimental sets in *S*. *hispanica*. The line across the box-plot depicts the median. Lower and upper boxes represent 25^th^ and 75^th^ percentiles. Whiskers represent the lowest and highest Cq values respectively.

The RNA isolated from different tissues was reverse transcribed into cDNA and used as template in qRT-PCR. The expression levels of 13 candidate reference genes in different tissue sets were examined using a SYBR Green-based qPCR assay, and the expression was determined using the quantification cycle (Cq) values. A low Cq value represents high expression level and vice-versa. The mean Cq values of candidate genes ranged from 19.65 to 28.62, with most falling in the range of 23 to 26. Across all the samples, 18S rRNA exhibited the lowest mean Cq value of 19.65 ± 1.13 (mean ± SD) indicating high expression, followed by GAPDH (20.6 ± 1.51) and EF1-α (22.87 ± 2.3), while PP2A (28.18 ± 2.2) and FtsH (28.62 ± 0.9) were the least expressed genes. The Cq value distribution of all genes in different samples are represented as a box-plot (Fig 2). The genes having high SD of Cq values exhibited more variable expression compared to those with lower SD. FtsH showed the smallest variation in gene expression (28.62 ± 0.9), while RCA showed most variable levels of expression (23.17 ± 4.5).

### Expression stability analysis of candidate reference genes

The stability values of reference gene(s) may vary from one experimental condition to the other. To obtain a reliable dataset of the reference genes under different experimental conditions, two most popular algorithms, geNorm and Normfinder were performed.

#### geNorm analysis

**Fig 3:**
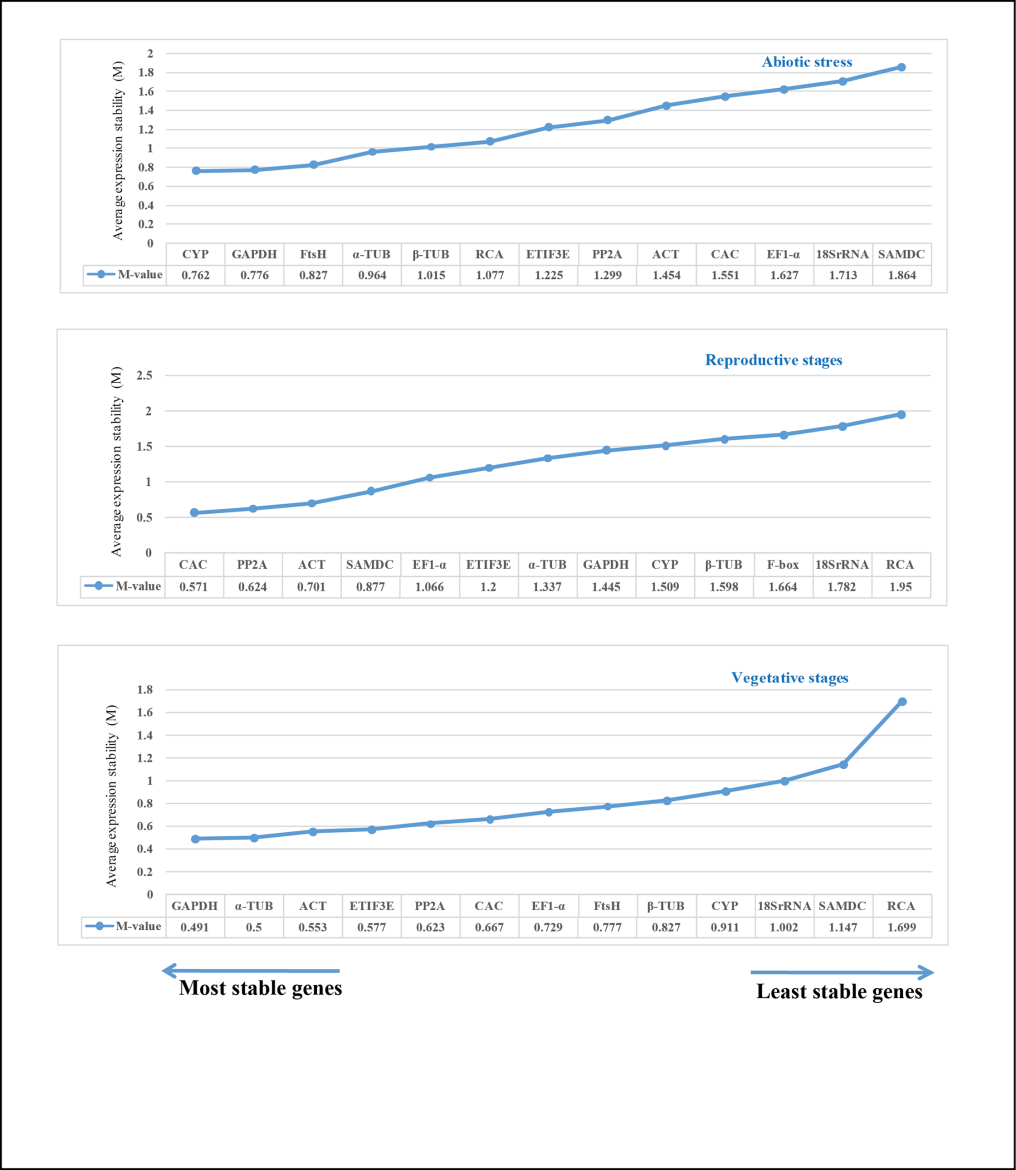
Gene expression stability (M) of 13 candidate reference genes across different experimental groups as assayed by geNorm. M-value is inversely related to gene stability. The cutoff M-value was set at 1.5; the direction of arrows indicate the most stable and least stable genes in the graph. The most stable genes are listed on the left and least stable genes are listed on the right.

geNorm analysis was performed to rank the 13 candidate reference genes by calculating expression stability value (*M*). The raw Cq values obtained for all genes were corrected according to their PCR efficiencies and then changed to relative quantities. These relative expression values were used in analysis to determine expression stability. *M*-value is negatively correlated to stability and the genes with lower *M*-value are more stable. geNorm recommends a cutoff *M*-value of 1.5 and the genes with *M*-value < 1.5 are considered as stably expressed. The expression stability ranking of tested genes in different tissue sets along with their *M*-values are listed in Fig 3. In abiotic stress, the lowest *M*-value was obtained for CYP and GAPDH (0.762 and 0.777 respectively) and they represent the genes with most stable expression, followed by FtsH (0.827) and α-TUB (0.965). EF1-α (1.614), 18S rRNA (1.701) and SAMDC (1.853) were the least stable genes based on the *M*-value. In reproductive stages, CAC and PP2A (*M*-value of 0.571 and 0.624) were the most stable genes while 18S rRNA and RCA with *M*-values of 1.772 and 1.95 were least stable. In vegetative stages, GAPDH and α-TUB (0.491 and 0.5) were the most stable genes while RCA (1.699) was least stable. 12 genes had a *M*-value of less than 1.5 and thus performed well in vegetative stages.

**Fig 4:**
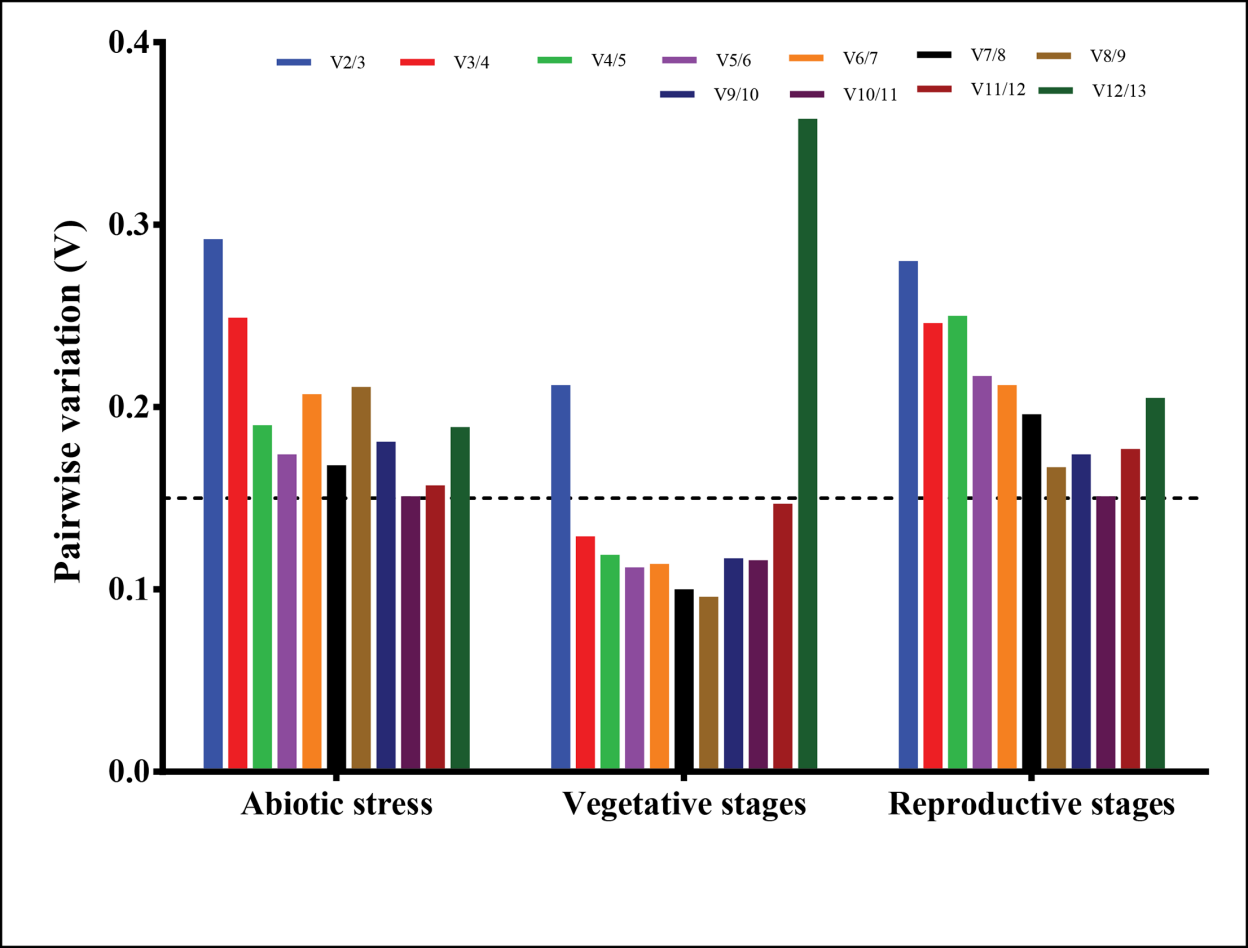
Optimal number of references genes required for normalization across different experimental sets as calculated by geNorm. Dotted line indicates cut-off value of 0.15, below which additional reference genes are not necessary for normalization.

The Vn/n+1 between normalization factors calculated by geNorm will help to determine the optimal number of reference genes needed for accurate normalization. A Vn/n+1 value <0.15 indicates that introducing an additional gene will make no significant contribution to normalization [8]. In the vegetative group, the V3/4 value was less than 0.15 (0.135), which suggested that three genes are sufficient for normalization. In the abiotic stress and reproductive group, the lowest pairwise variation value was above 0.15 (Fig 4).

#### Normfinder analysis

Normfinder is a Microsoft Excel^TM^ based program that requires the input data to be given on a linear scale. Therefore, the raw Ct values obtained for each gene should be transformed into relative expression levels according to the given formula 2^- ΔCt^ (ΔCt = Ct value of each sample—minimum Ct value).

**Fig 5:**
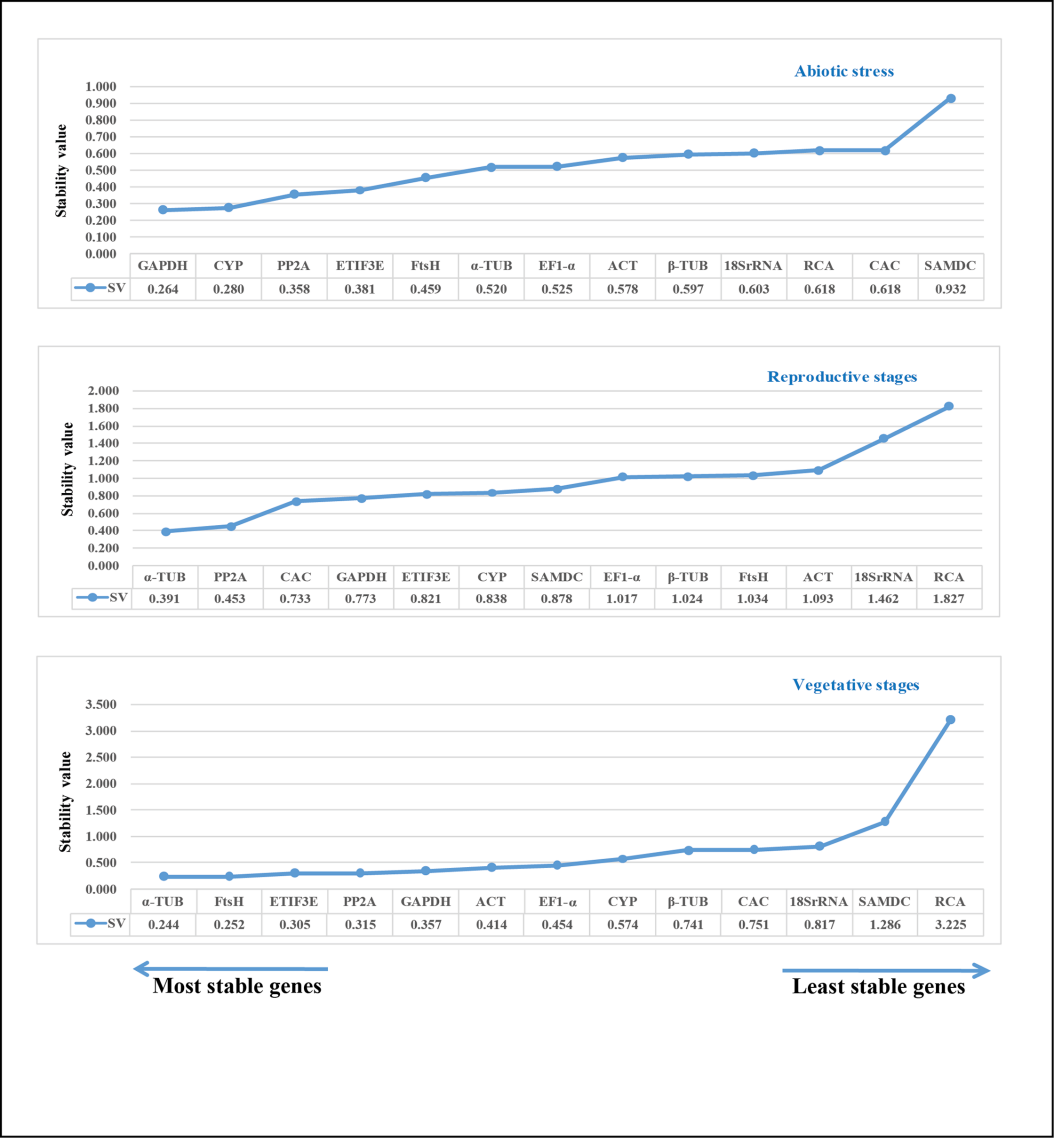
Stability values of 13 candidate reference genes across different experimental groups as assayed by Normfinder. Lower stability values indicate highly stable genes and higher stability values indicate less stable genes. The direction of the arrows indicate the most stable and least stable genes in the graph. The most stable genes are listed on the left and least stable genes are listed on the right.

These values are then imported into Normfinder software (https://moma.dk/normfinder-software) to analyze gene expression stability. It measures the stability based on intra and inter-group expression variations, and a low stability value indicates stable gene expression. The gene stability rankings generated by Normfinder are shown for each group and the stability values are also included in Fig 5. In the abiotic stress group, CYP (stability value 0.264) and GAPDH (0.278) were ranked the most stable genes (also ranked similarly by geNorm), while SAMDC (0.929) was the least stable gene. In the reproductive group, α-TUB (0.389) and PP2A (0.451) emerged as the most stable genes, followed by CAC (0.730) while geNorm ranked α-TUB at seventh place. 18S rRNA (1.464) and RCA (1.829) were the two least stable genes, as with the geNorm analysis. In the vegetative group, FtsH (0.253) and α-TUB (0.255) occupied the top positions, while geNorm ranked FtsH at a medium position. Finally, Normfinder also reported SAMDC and RCA as the least stable genes in accordance with geNorm.

### Validation of the best and least ranked reference genes

**Fig 6:**
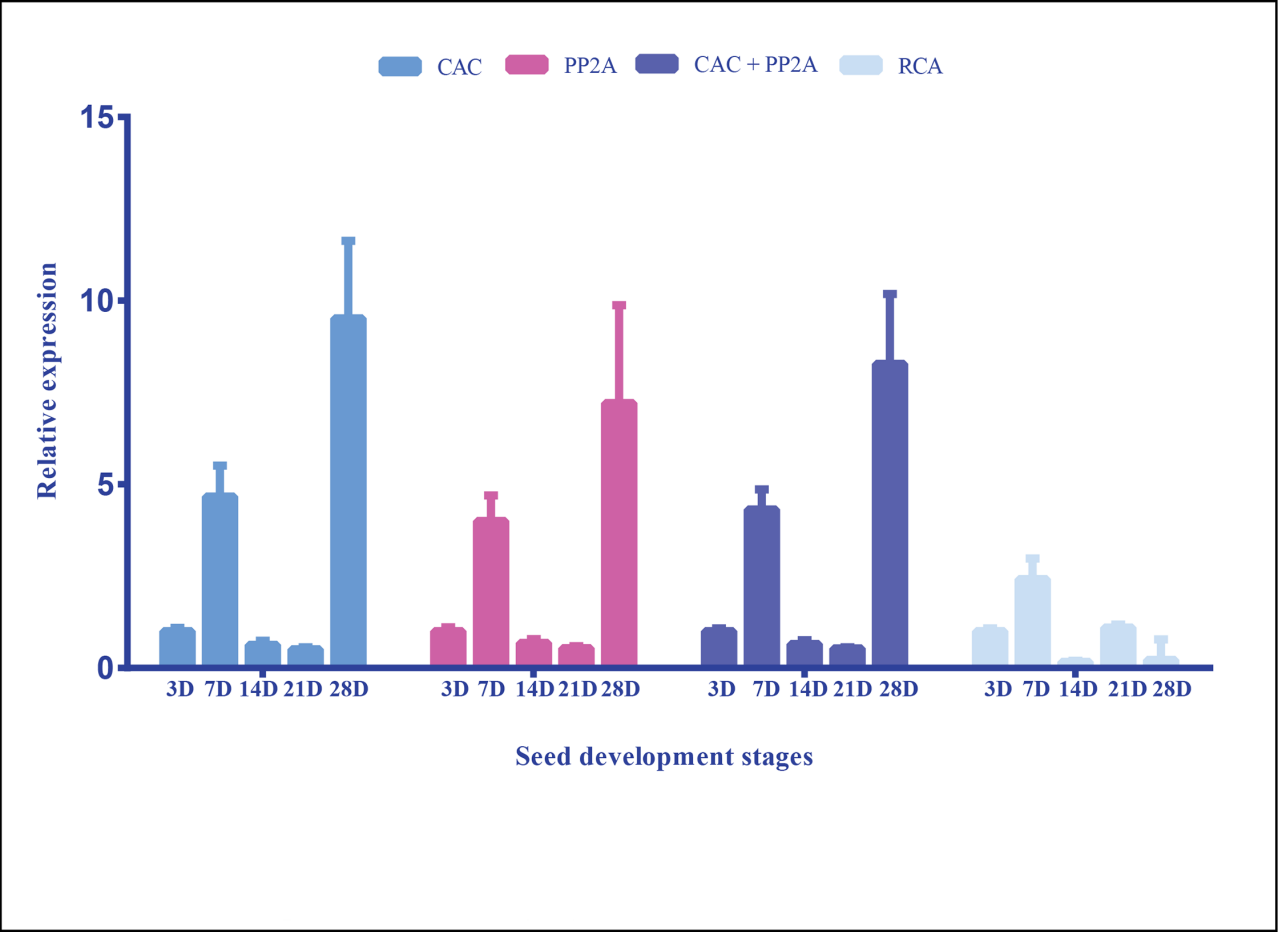
Validation of the reference genes in reproductive stage group. The relative expression levels of DGAT1 was normalized by using the two most stable genes, PP2A and CAC in combination and individually, and also with the least stable gene, RCA. D represents DAF–Days after flowering. Values shown are relative expression levels ± SD (n = 3).

To validate the genes identified in the current study, we checked the expression levels of a key enzyme, DGAT1 in different stages of seed development (reproductive stages). Triacylglycerols are the major storage forms of energy and have crucial roles in seed oil accumulation, germination and seedling development. DGAT1 plays a crucial role in the final step of TAG synthesis by catalyzing final acylation in pathway of diacylglycerol (DAG) to form TAG [36]. Overexpression of DGAT1 has been performed in different plant species and this has enhanced the TAG content [37, 38]. It is considered the rate-limiting enzyme in TAG accumulation, and is being exploited to increase the oil content through genetic engineering in oil plants and microalga for economic benefit [36, 39]. The expression of DGAT1 was normalized using the two best genes, PP2A and CAC in combination and individually. For both genes, the transcript abundance increased at second stage (7DAF) compared to first stage (3DAF), and finally increased to a maximum at the final stage of seed maturation. When PP2A and CAC were used in combination, the same pattern was seen. However, normalization with the least reported stable gene, RCA, the expression pattern was completely changed (Fig 6). The results highlight the importance of validated reference genes for target normalization.

## Discussion

Currently, qRT-PCR is considered as the most appropriate method for gene expression profiling due to its high sensitivity, accuracy and reproducibility [40]. Precise measurement of target gene expression requires the use of suitable reference genes that are stably expressed. However, random or inappropriate selection of reference genes for normalization may lead to misinterpretation of results [41]. Recent reports have shown that the expression of commonly used reference genes varies with different experimental conditions. Therefore, it is mandatory to validate reference genes for each experimental condition in different species [15]. Hence, the aim of the study was to identify ideal reference genes with stable expression levels under different experimental conditions in *S*. *hispanica*.

We checked the expression levels and stability of 13 candidate reference genes across different tissue samples, i.e. vegetative stages, reproductive stages and six abiotic stress treatments in *S*. *hispanica*. Two softwares namely, geNorm and Normfinder were used to identify the stable reference genes in the studied experimental conditions. As the two algorithms operate differently, the results were slightly different in some conditions. For example, in reproductive stages, geNorm ranked CAC and PP2A as the two most stable genes while Normfinder ranked α-TUB and PP2A as the best genes. α-TUB was ranked at a medium position in geNorm, however CAC was ranked third in Normfinder. In vegetative stages, FtsH was ranked at a medium position in geNorm but was ranked second in Normfinder. The gene α-TUB was ranked top in both algorithms. However, both geNorm and Normfinder recommended CYP and GAPDH as the suitable reference genes in abiotic stress treatments. In addition, the two least stable genes for each set were the same in geNorm and Normfinder. The apparent divergence in ranking is due to the discrepancies in the two algorithms to measure stability [42]. The geNorm works upon stepwise exclusion of the least stable genes, which is based on the expression stability value (*M*) [8]. Normfinder, an excel based tool takes inter- and intra-group variations into account and combines them into a stability value [7]. The difference in the ranking by the two algorithms was also observed in many other studies [14, 43–45].

Our results are in accordance with previous studies, wherein CYP was reported to be the stably expressed gene under abiotic stress conditions in peanut [29], sorghum [21] and salt-stressed *S*. *tuberosum* [11]. The CYP gene also performed well in switchgrass [46]. Similarly, GAPDH was reported to be a stably expressed gene under stress conditions in chickpea [13], tall fescue [44], heat and salinity stress in pigeon pea [31], and hormone stimuli in parsley [14]. In contrast, GAPDH was shown to be a least stable gene in *B*. *juncea*. SAMDC, the least stable gene in abiotic stress treatment was reported to be unstable in switchgrass [46] and 18S rRNA was also reported as unstable in Jute [42] and oxytropis [47]. Further, PP2A was found to be stably expressed in maturing *B*. *napus* embryos [48], developmental stages in *A*. *thaliana* [6] and life stages in Striga [49]. CAC was also reported to be the stable gene during development in *B*. *juncea* [22] and *B*. *arvensis* [50]. Similar to the present study, RCA was also reported to be the least stable gene in reproductive development in papaya [51].

Data normalization with a single reference gene may give rise to erroneous results [52] and thus usage of two or more reference genes has become the common way. geNorm has suggested to use three reference genes for vegetative stages as the V3/4 value was less than 0.15. For reproductive stages and abiotic stress, the value was above 0.15. However, the V (pairwise variation) value of 0.15 should not be considered as an absolute one and high V values have been reported in few species. [53–55]. Vandesompele et al., 2002 have proposed that minimum three reference genes are necessary for precise normalization [8]. However, the usage of multiple reference genes may lead to misinterpretation of results.

DGAT1, a key enzyme involved in triacylglycerol synthesis was used to validate the reference genes identified in the study. DGAT catalyzes the synthesis of TAG from diacylglycerol and is pivotal to oil accumulation. The two best reference genes, CAC and PP2A were used alone and in combination for normalization. With both genes, there was an increase in transcript expression at 7DAF (second stage) compared to 3DAF (first stage) and then finally increased in the final stage (28DAF). When normalized using the least stable gene RCA, the expression pattern was not conclusive.

In conclusion, we evaluated the expression stabilities of 13 candidate reference genes in different abiotic stresses (NaCl, PEG, SA, ABA, heat, and cold), vegetative and reproductive stages of *S*. *hispanica*. Two statistical algorithms, geNorm and Normfinder were used to assess the stability of reference genes, and some slight variation was observed because of the way both algorithms operate to calculate stability. Overall, we propose that in *S*. *hispanica*, CYP and GAPDH can be used for abiotic stresses; PP2A and CAC for reproductive development, α-TUB, ETIF3E and PP2A for vegetative stages. In addition, the expression pattern of DGAT1 was analyzed using the best reference genes identified in the study. The reference genes identified will benefit future studies of gene expression in various tissues of S. *hispanica*.

## Supporting information

S1 File RNA integrity values determined by Bioanalyzer. (PPTX)

S2 File Melt curves of 13 genes under different experimental conditions. (PPTX)

S3 File Amplification efficiencies of primers used in the study. (PPTX)

S1 Table Annealing temperatures of primers used in the study. (DOCX)

S2 Table Sequences of the PCR amplicons. (DOCX)
