# Supplementary figures and images for: Selection and validation of appropriate reference genes for quantitative real-time PCR analysis in Salvia hispanica
Source: PLoS One. 2017 Nov 1;12(11):e0186978. doi: 10.1371/journal.pone.0186978 (PMC5665522; doi:10.1371/journal.pone.0186978)

## Slide 1
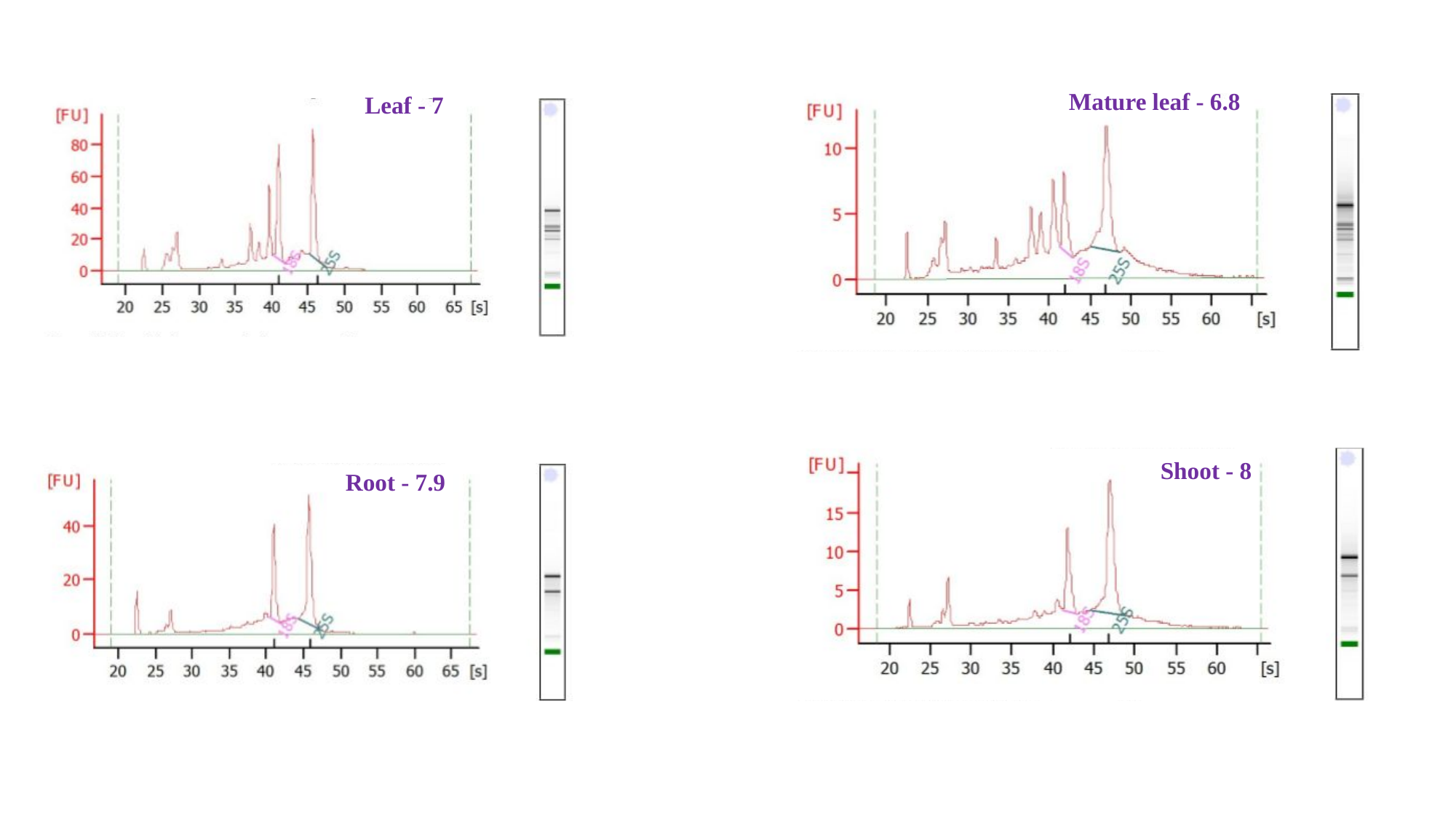

Mature leaf - 6.8
Leaf - 7
Shoot - 8
Root - 7.9

## Slide 2
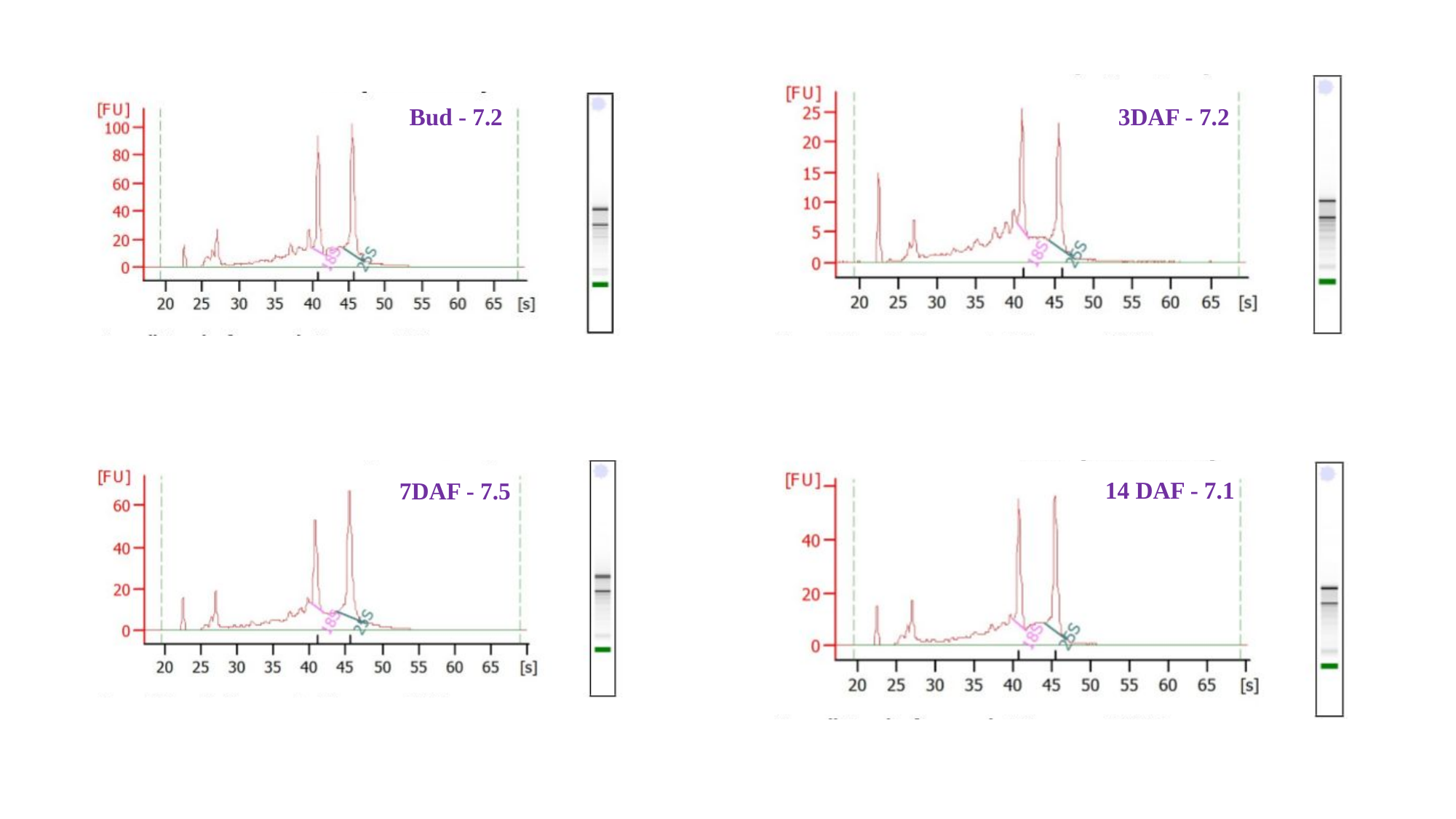

3DAF - 7.2
Bud - 7.2
14 DAF - 7.1
7DAF - 7.5

## Slide 3
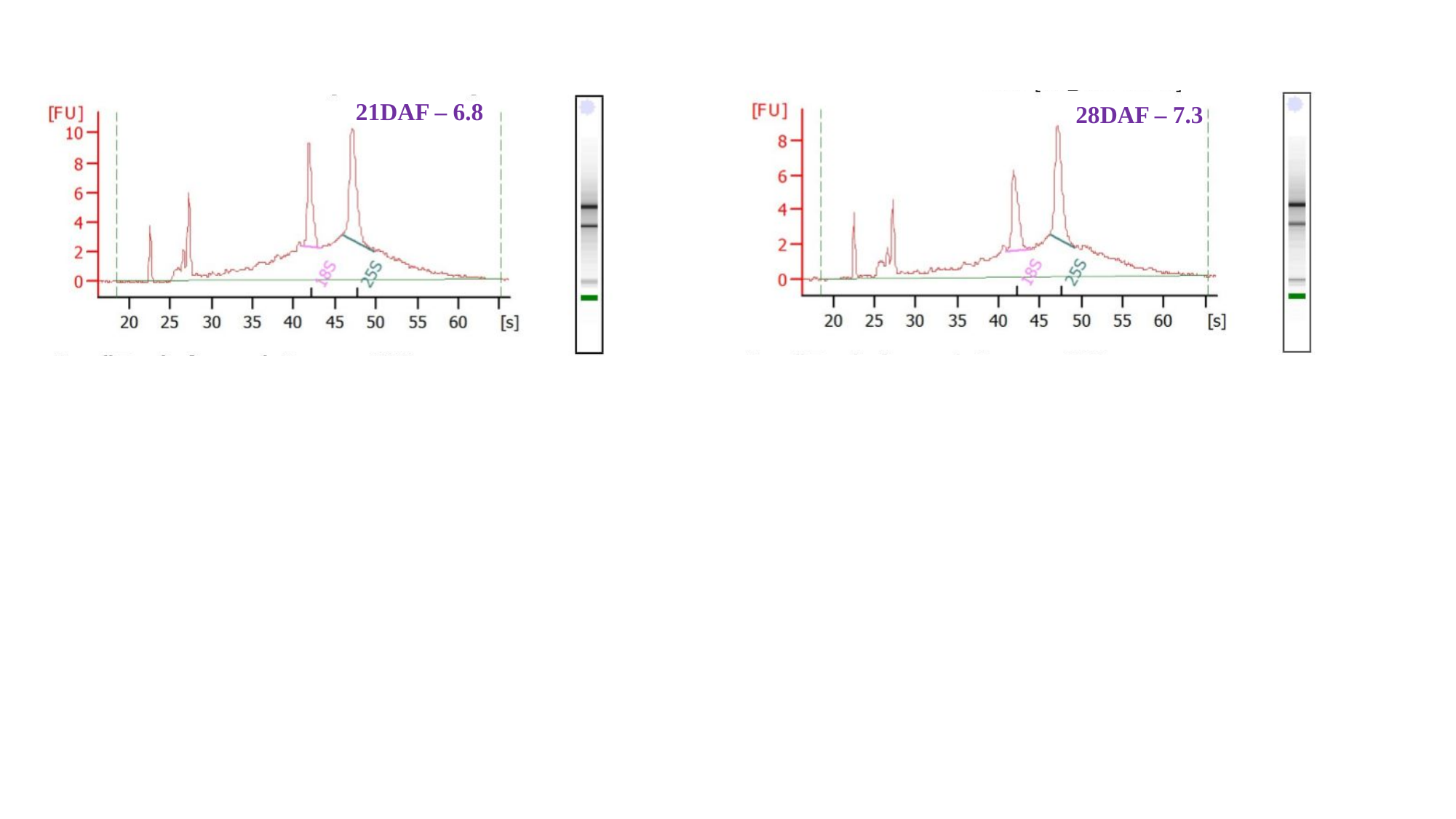

21DAF – 6.8
28DAF – 7.3

Supplement: S1 File — (PPTX) [file pone.0186978.s001.pptx]

## Slide 1
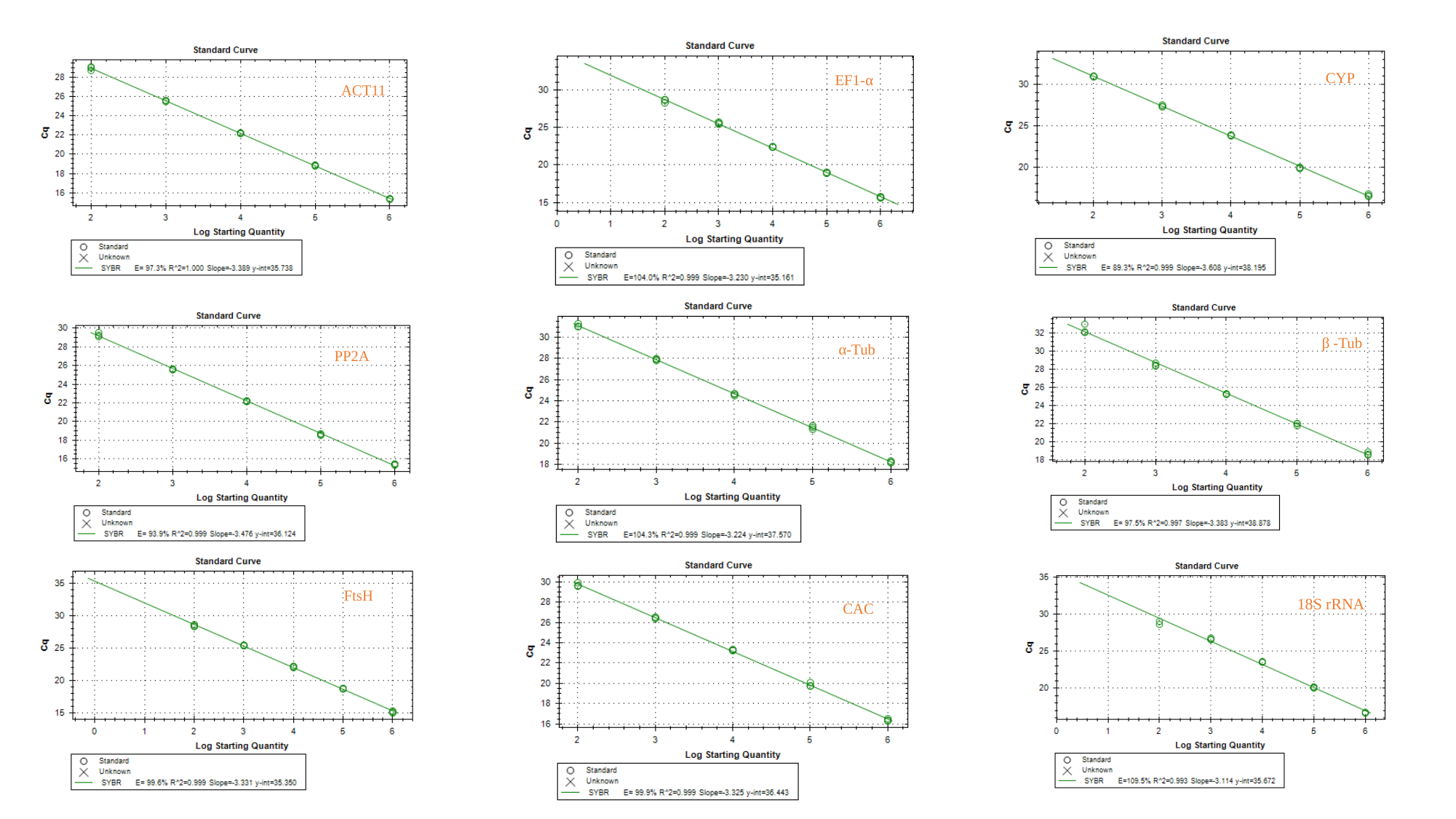

CYP
EF1-α
ACT11
β -Tub
α-Tub
PP2A
FtsH
18S rRNA
CAC

## Slide 2
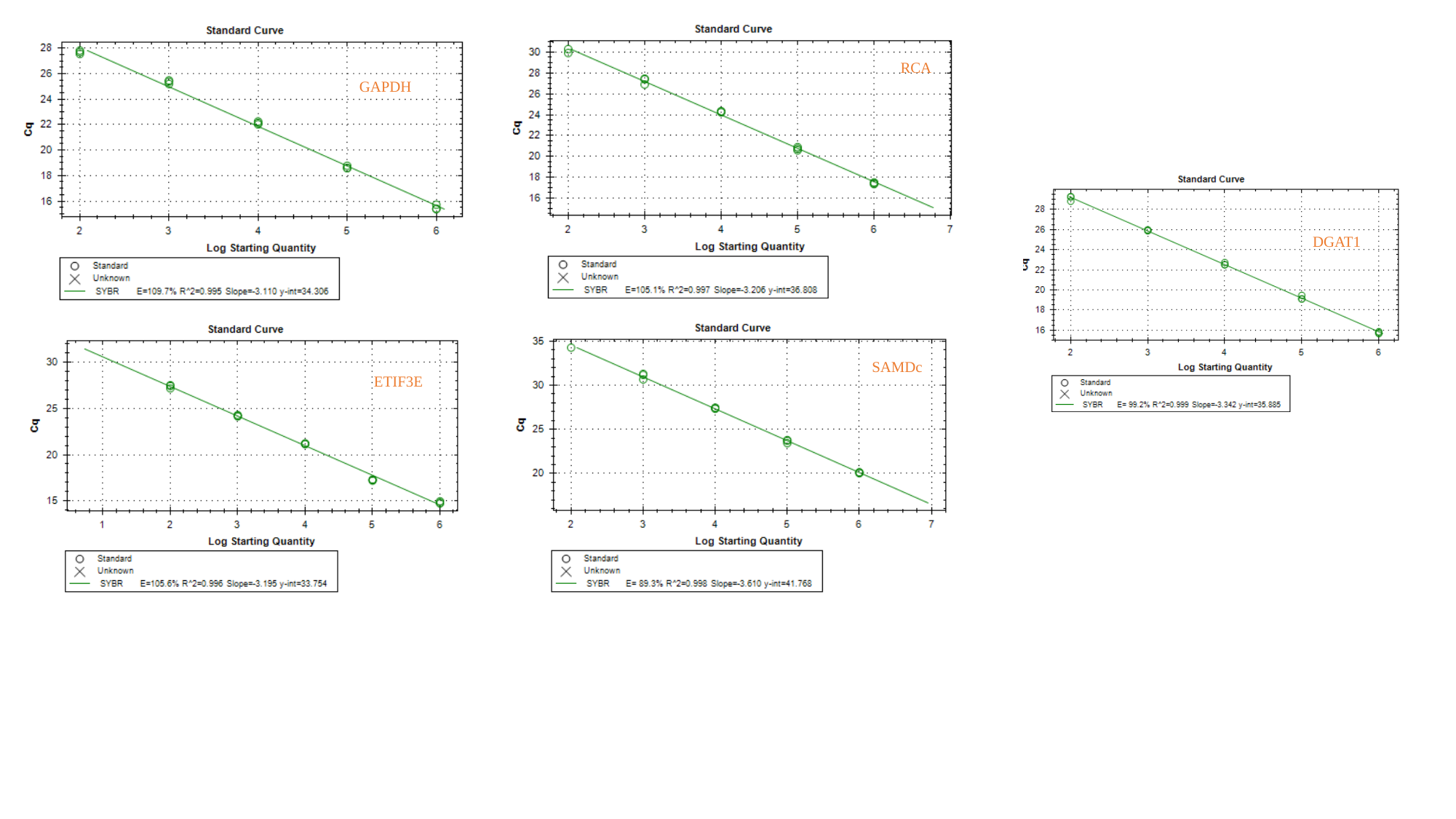

RCA
GAPDH
DGAT1
SAMDc
ETIF3E

Supplement: S3 File — (PPTX) [file pone.0186978.s003.pptx]
