# Supplementary material for: Selection and validation of appropriate reference genes for quantitative real-time PCR analysis in Salvia hispanica
Source: PLoS One. 2017 Nov 1;12(11):e0186978. doi: 10.1371/journal.pone.0186978 (PMC5665522; doi:10.1371/journal.pone.0186978)
Supplement: S2 File — (PPTX) [file pone.0186978.s002.pptx]

## Slide 1
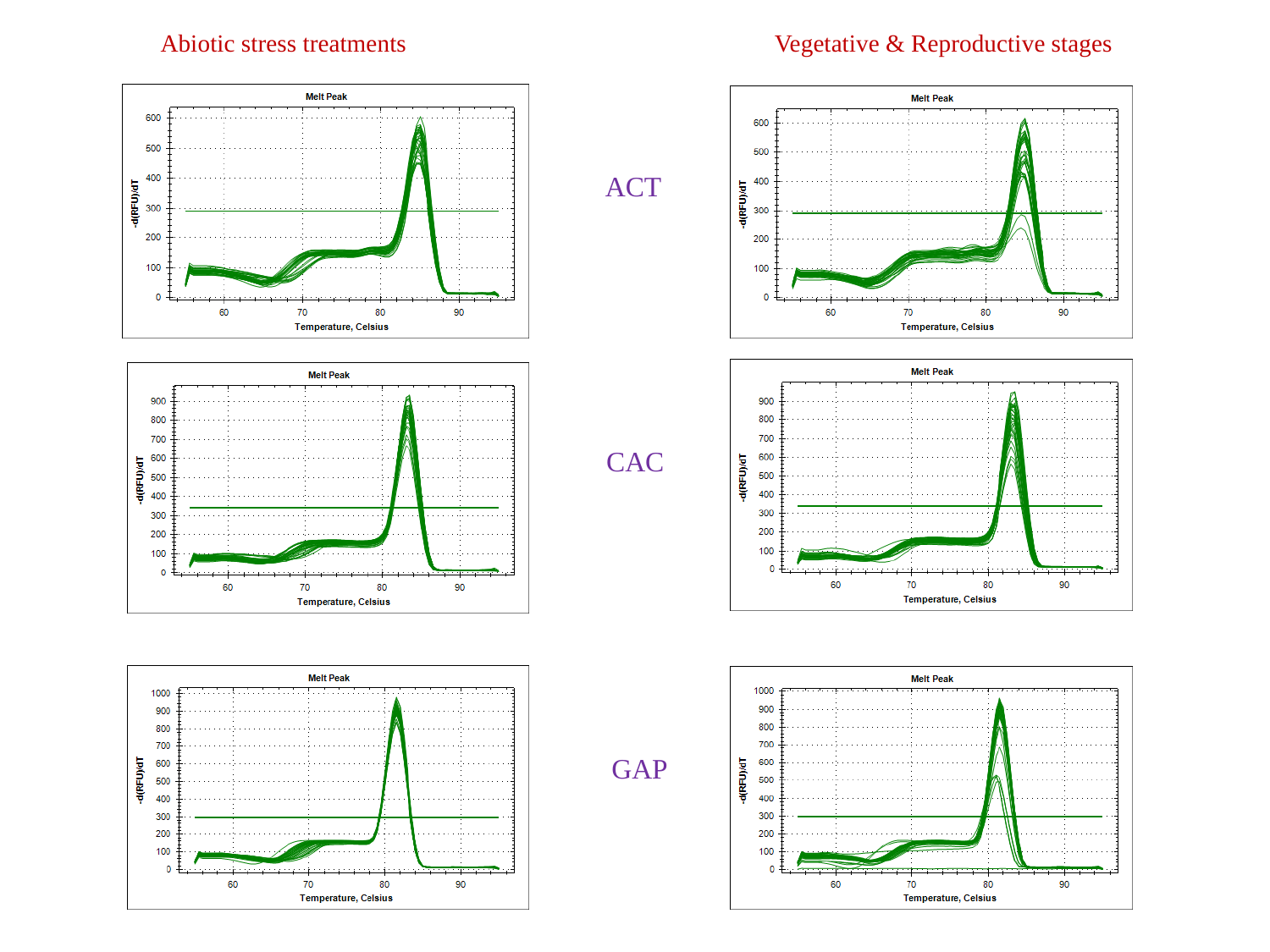

Abiotic stress treatments
Vegetative & Reproductive stages
ACT
CAC
GAP

## Slide 2
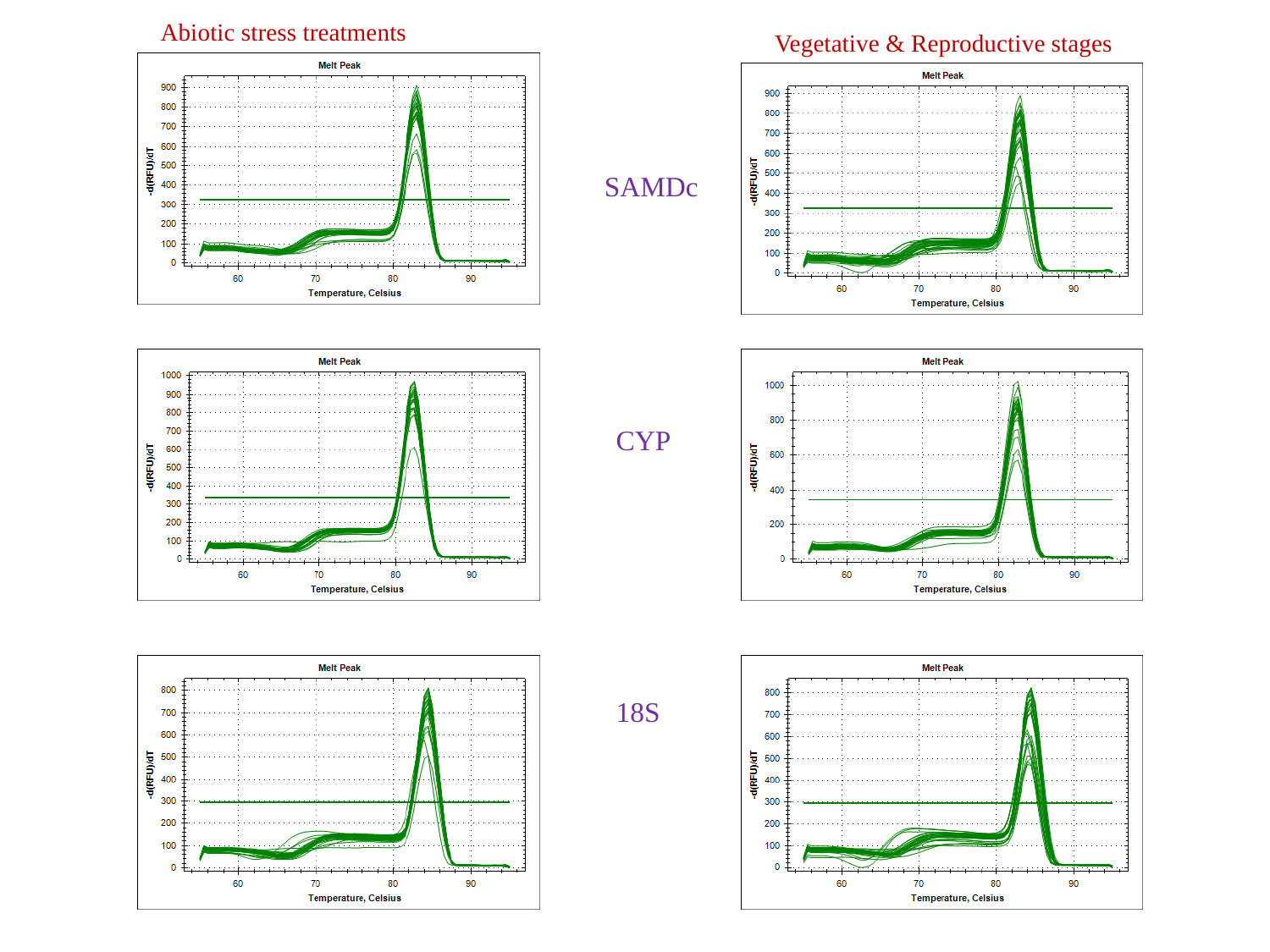

Abiotic stress treatments
Vegetative & Reproductive stages
SAMDc
CYP
18S

## Slide 3
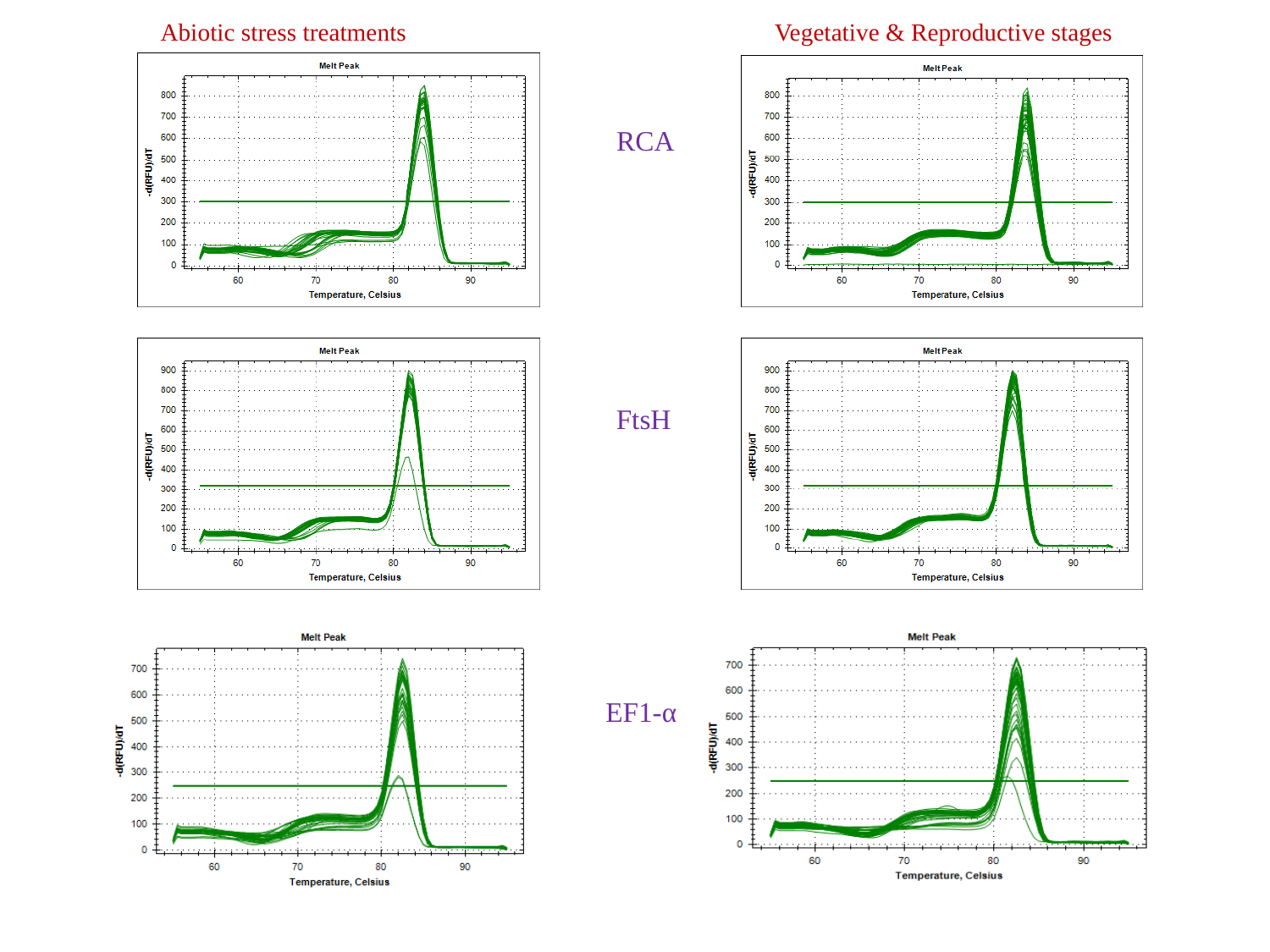

Vegetative & Reproductive stages
Abiotic stress treatments
RCA
FtsH
EF1-α

## Slide 4
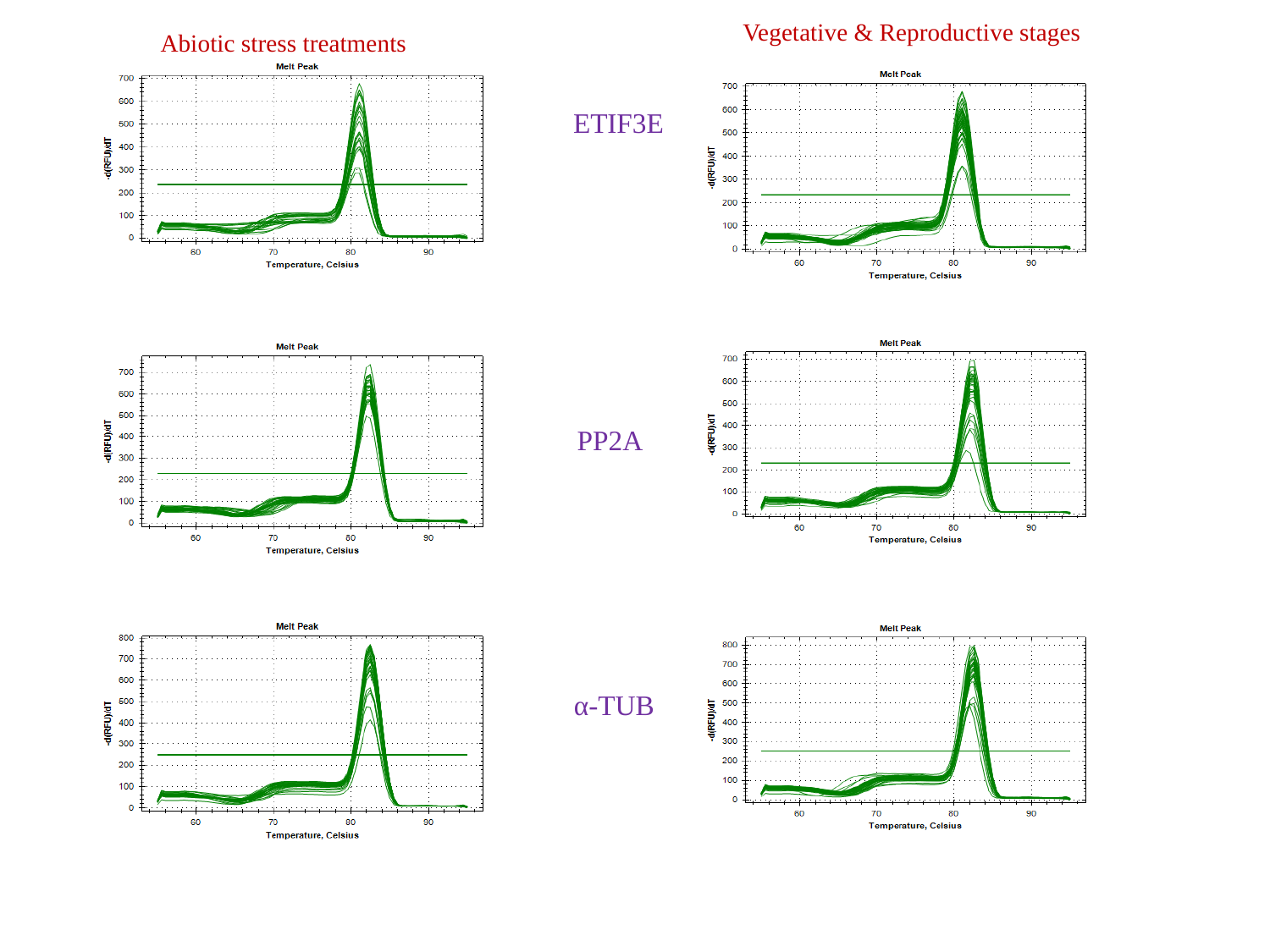

Vegetative & Reproductive stages
Abiotic stress treatments
ETIF3E
PP2A
α-TUB

## Slide 5
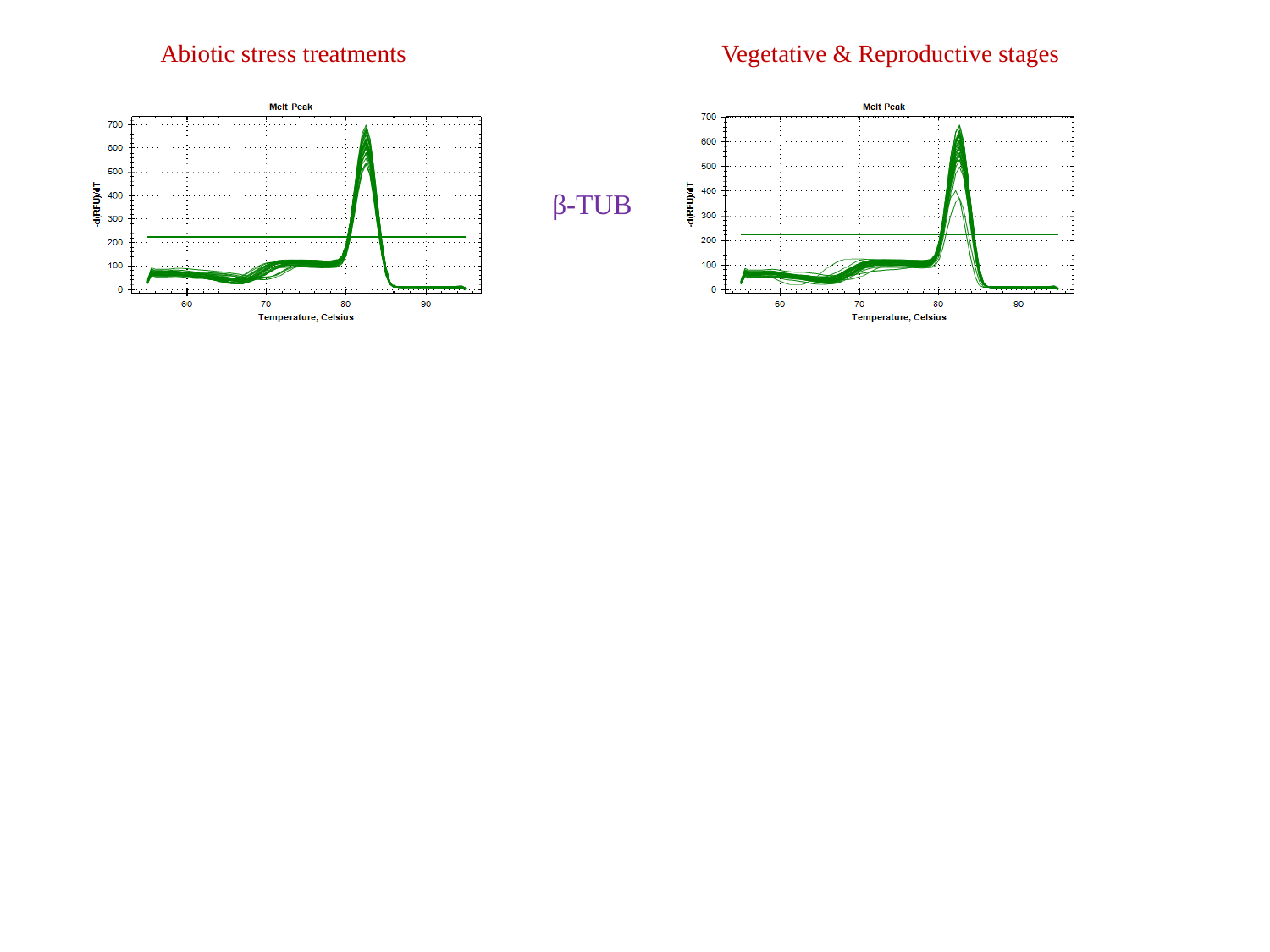

Abiotic stress treatments
Vegetative & Reproductive stages
β-TUB
