# Supplementary material for: Selection and validation of appropriate reference genes for quantitative real-time PCR analysis in Salvia hispanica
Source: PLoS One. 2017 Nov 1;12(11):e0186978. doi: 10.1371/journal.pone.0186978 (PMC5665522; doi:10.1371/journal.pone.0186978)
Supplement: S1 Table — (DOCX) [file pone.0186978.s004.docx]

**S1 Table.** A gradient was run from 55-65ᵒC on qRT-PCR machine and the optimal annealing temperature for each primer pair was taken for analysis.

| **S. No** | **Gene Name** | **Tm (ᵒC)** |
| --- | --- | --- |
| 1 | Actin11 | 61.4 |
| 2 | Elongation factor-1alpha | 63.3 |
| 3 | Eukaryotic translation Initiation factor 3E | 59.0 |
| 4 | Alpha tubulin | 63.3 |
| 5 | Beta tubulin | 61.4 |
| 6 | Glyceraldehyde-3-phosphate dehydrogenase | 57.0 |
| 7 | Clathrin adaptor complex | 61.4 |
| 8 | Serine/threonine-protein phosphatase | 61.4 |
| 9 | Rubisco activase | 61.4 |
| 10 | FtsH protease | 61.4 |
| 11 | Cyclophilin | 61.4 |
| 12 | S-adenosyl methionine decarboxylase | 63.3 |
| 13 | 18S ribosomal RNA | 61.4 |
