# Supplementary material for: Selection and validation of appropriate reference genes for quantitative real-time PCR analysis in Salvia hispanica
Source: PLoS One. 2017 Nov 1;12(11):e0186978. doi: 10.1371/journal.pone.0186978 (PMC5665522; doi:10.1371/journal.pone.0186978)
Supplement: S2 Table — (DOCX) [file pone.0186978.s005.docx]

**S2 Table. Sequences of the PCR amplicons used in the current study**

| ACT | ACTGGAATGGTCAAGGCTGGGTTTGCTGGGGATGATGCTCCGAGAGC  TGTCTTCCCGAGTATAGTGGGGCGCCCTCGCCACACTGGAGTCATGGTTGGGATGGGCCAGAAAGA |
| --- | --- |
| EF1-α | CTGTCCAGGAGCCAAAGAGGCCCTCAGACAAGCCTCTCCGTCTCCCACTTCAGGATGTCTACAAGATTGGTGGTATTGGAACTGTGCCAGTCGGAAGAGTTGA |
| ETIF3E | TGTGGAAACTACTCCGGTGCTGCTGATTATCTGTATCAGTACAGAGCCTTGTGCACCAACAGTGACAAGAGTTTGAGTGCATTGTGGGGAAAATTGGCAGCAG |
| α-TUB | CTCGCGCATTGACCACAAATTCGACCTCATGTACTCCAAGAGAGCGTTTGTTCACTGGTACGTCGGTGAGGGAATGGAGGAAGGTGAGTTCAGCGAAGCTC |
| ß-TUB | GTACACCGGGGAAGGAATGGACGAGATGGAGTTCACTGAAGCAGAGAGTAACATGAACGACCTGGTGGCCGAGTACCAGCAATATCAAGATGCGACTGCTGACGAA |
| GAPDH | TCAAGGAGGAGTCTGAGGGAAATCTGAAGGGAATTCTAGGCTACACCGAAGATGATGTTGTGTCTACTGATTTTGTCGGCGACAACAGGTCAAGCATTTTCGATGCCAAGGCTGGAATTG |
| CAC | CGGCTTCCGCGATTTACTTCCTTAATCTTCGCGGCGATGTTCTCATCAATCGCCTCTACCGTGACGACGTCGGAGGCAACATGGTGGATGCTTTTCGAGTGC |
| PP2A | GGCGATATCCACGGACAGTTTTACGATCTGATCGAGCTGTTCCGGATTGGCGGCAATGCTCCTGATACAAATTATCTCTTCATGGGGGACTACGTCGACCGTGGGTACTATTCGG |
| RCA | CCGAAGATGACGCTGGAGAAGCTGCTGCAGTACGGGAACATGCTGGTGGCGGAGCAGGACAATGTGAAGAGAGTGCAGTTGGCTGACAAATACTTGAAGGACGCAGCTCTT |
| FtsH | GCGCCAAAGCAGTGAGTATGGCTGATCTTGAGCATGCCAAGGATAAAATCGTGATGGGAAGCGAGCGCAAATCTGCAGTTATATCTGATGAATCACGCAGGAATACAGCCTACCACGAG |
| CYP | ACCAACCATTTCTTCCGGGTAGACAAGGGGTTCGTCGCCCAAGTTGCTGATGTTGGTGGAGGAAGAACTGCTCCAATGAATGAAGTGCAGAGGTTGGAAGCTGAG |
| SAMDc | GCTTCATATTCCCGGGTGCTCAGCCATTCCCCCACCGGAGCTTCAACGAAGAAGTTGCTGTTCTTGACGACCATTTCTCCAAACTTGGACTGATGAGCGAAGCCTATGTGATGGGA |
| 18SrRNA | CGGGTGACGGAGAATTAGGGTTCGATTCCGGAGAGGGAGCCTGAGAAACGGCTACCACATCCAAGGAAGGCAGCAGGCGCGCAAATTACCCAATCCTGACACGGGGAGGTA |
